# Supplementary figures and images for: The SKMT Algorithm: A method for assessing and comparing underlying protein entanglement
Source: PLoS Comput Biol. 2023 Nov 27;19(11):e1011248. doi: 10.1371/journal.pcbi.1011248 (PMC10703313; doi:10.1371/journal.pcbi.1011248)

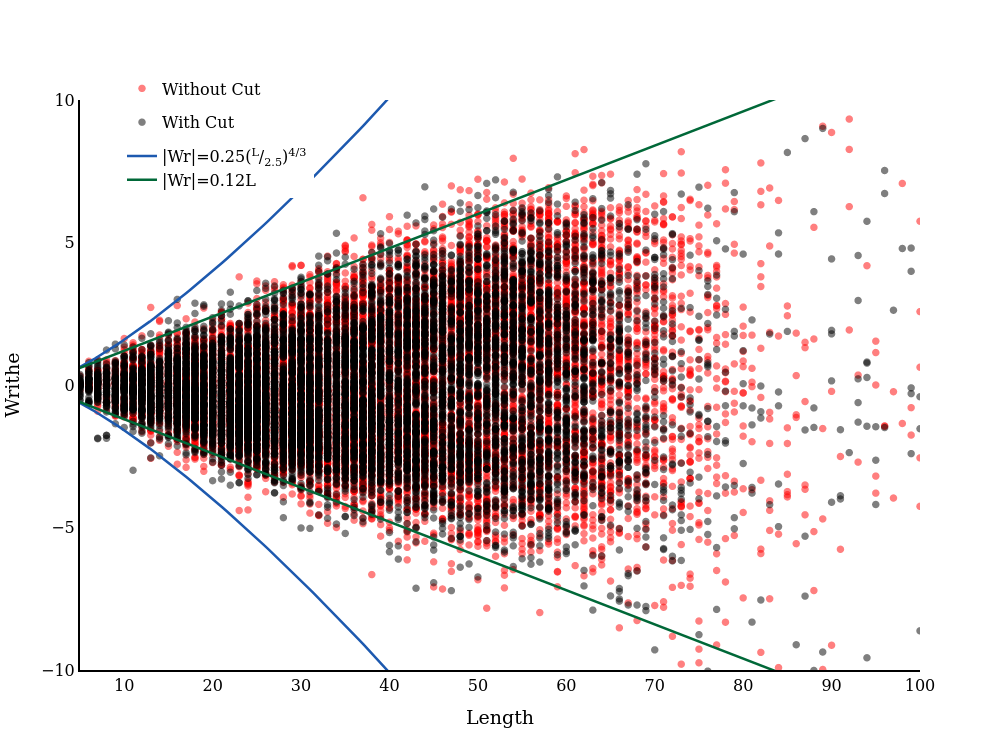

Supplement: S1 Fig — It is often the case that the N terminus may be missing from a PDB entry, or a purification tag is used to experimentally determine the structure. To study the potential effect of these foibles on our writhe calculations, we randomly cut between 10 − 20 residues from the start of each PDB file before applying the SKMT smoothing. The writhe of the SKMT backbones are calculated as before and their values are plotted against the length (number of points) of the smoothed curve. In S1 Fig. The distribution is overlaid with the standard distribution of writhe presented in Fig 6 in red for comparison. The overall shape of the two distributions is the same, with the majority of points falling well within the linear bound. Indeed, with the cut residues we find that 98.3% of the data falls within the linear bound, compared with 97.9% in the original case. Since the cutting of 10–20 residues from the N terminus end of each protein means removing 1 or 2 points from the SKMT curve, there is little change to the nature of the overall entanglement. (TIF) [file pcbi.1011248.s001.tif]

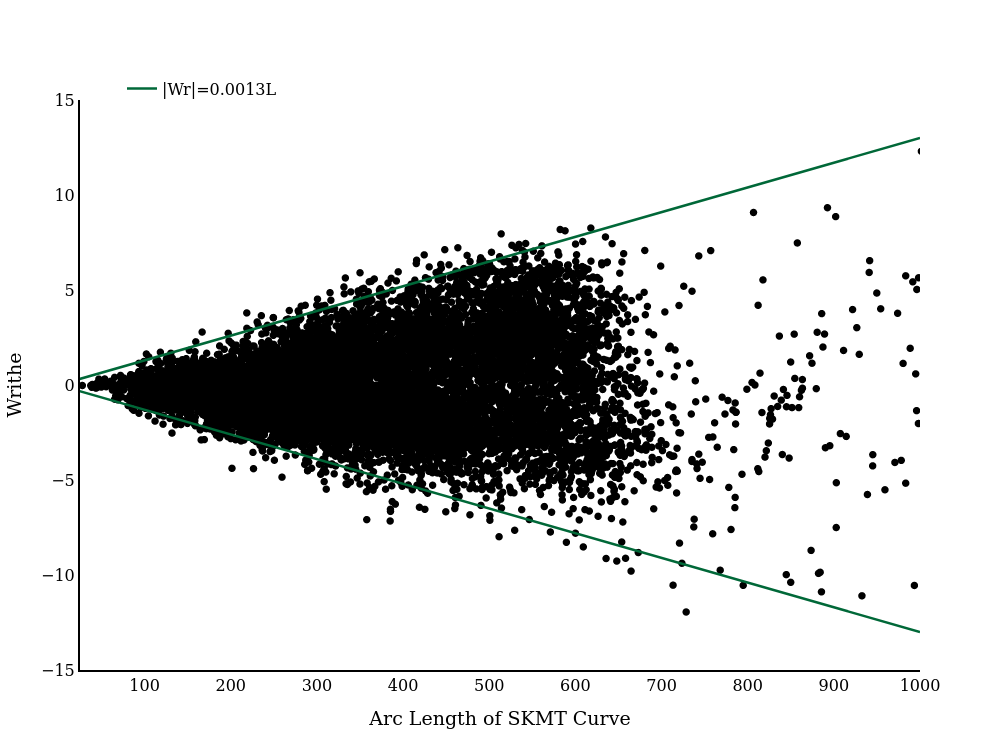

Supplement: S2 Fig — In S2 Fig we plot the distribution of writhe of the SKMT smoothed backbones against their arclength. Since this definition of length is proportional to our choice of length, the empirically determined linear bounding curve performs similarly well for this distribution containing 98.1% of the data. However, it appears there is more of a gap between the bounding line and the main distribution for SKMT curves of arclength between 150–250, compared to the equivalent section of the plot in the main text (Fig 6). In some sense the bound appears slightly less “tight”. As a heuristic measure of the tightness of the linear bound, we compute the distance from the linear bound for the closest value at each length, then take the average of these distances. In the case of the arclength distribution, this average closest distance is 3.1 whereas for the SKMT length distribution it is just 1.2, indicating that our choice of length better captures the length scaling relationship of entanglement we are aiming for in this study. (TIF) [file pcbi.1011248.s002.tif]

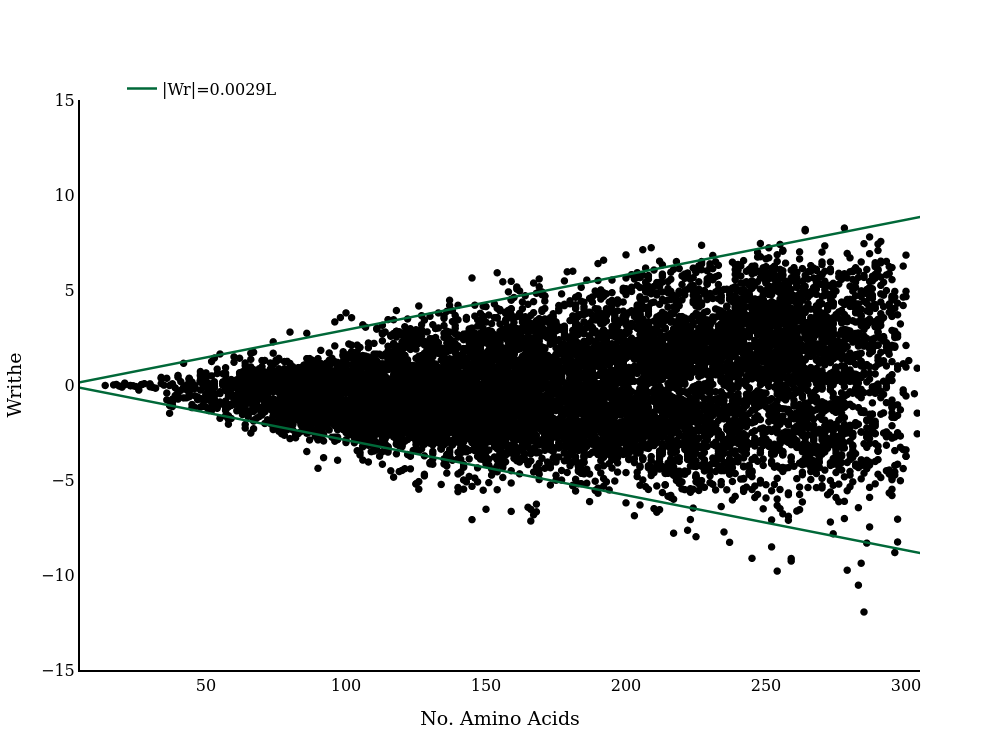

Supplement: S3 Fig — In S3 Fig we plot the distribution of writhe of the SKMT smoothed backbones against the number of amino acids of the respective protein. An empirically determined linear bounding curve fit to contain the same proportion of the data as in Fig 6 is shown in green. The average closest distance to the bound for this fit is 2.9, indicating that the tightness of this fit is not optimal. (TIF) [file pcbi.1011248.s003.tif]

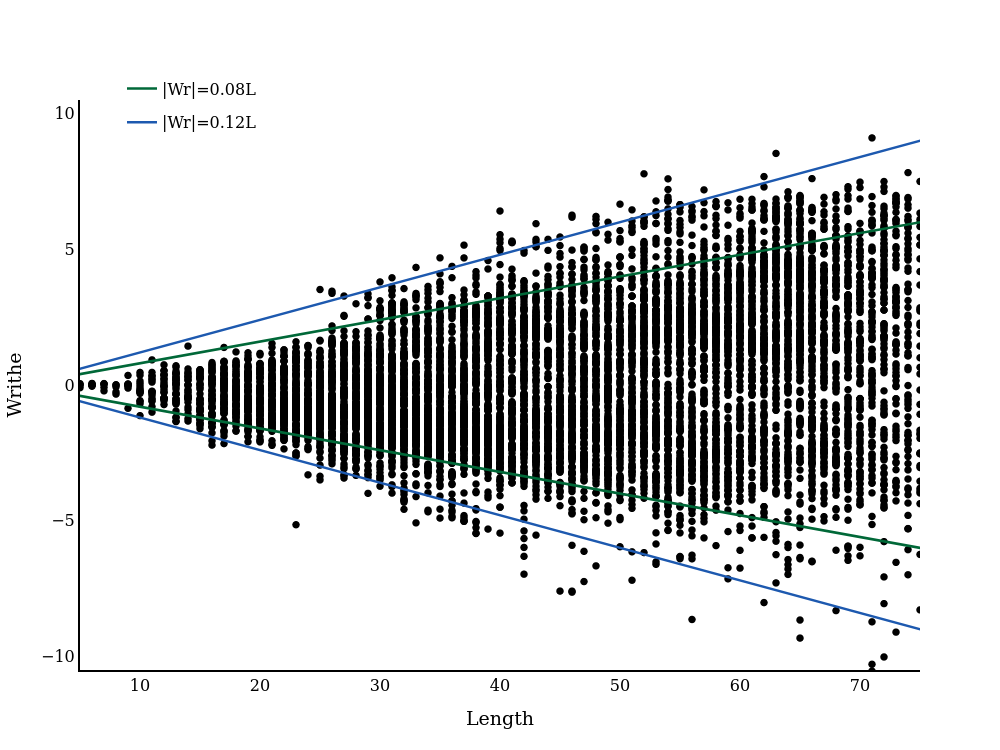

Supplement: S4 Fig — In S4 Fig we see that there is no clear linear relationship between writhe and length for backbones that are uniformly smoothed by sampling every n = 4th amino acid (a similar lack of clear relationship was obtained for n = 3, 5, 6, 7). In the SKMT case, a linear gradient of 0.12 is sufficient to capture 97.9% of the data, with a uniform spread of outliers across all lengths. Here, a linear gradient of 0.12 achieves a similarly good fit containing 98.8% of the data, however it is an overestimation for positively entangled small proteins, and negatively entangled large proteins, with an average closest distance in these regions of 4.6. In green, we see a linear bounding curve with gradient of 0.08. Though the average closest distance for this fit is good at 0.19, only 89.1% of the data lies within this bound. The SKMT smoothed approach therefore performs better at representing the relationship between potential complexity and secondary structure. (TIF) [file pcbi.1011248.s004.tif]

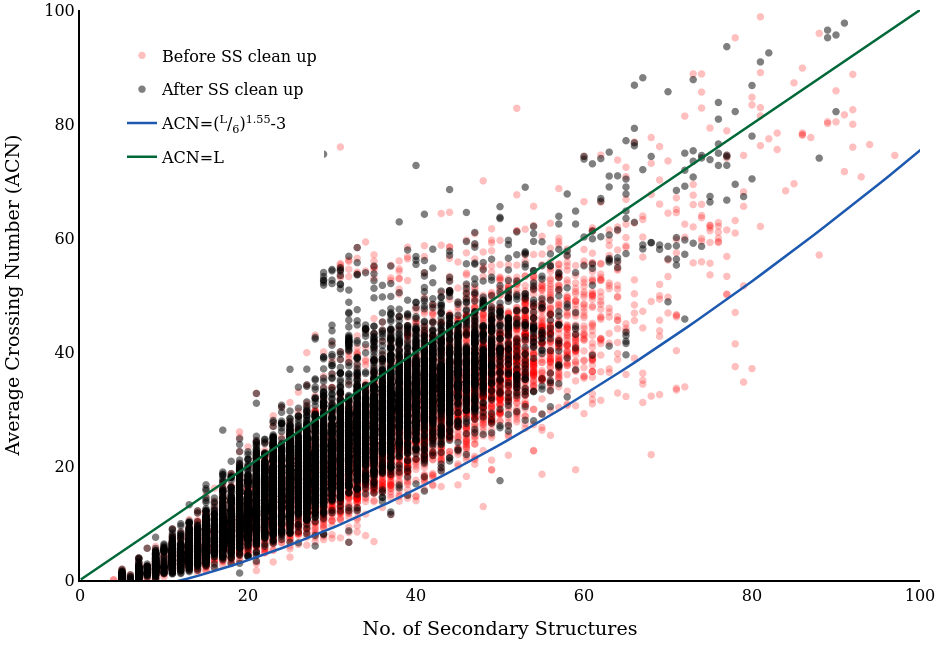

Supplement: S5 Fig — During the initial study some proteins had acn well below the empirically determined lower bound despite appearing maximally entangled. This was due to the fact that our definition length in this study is dependent on the number of SSEs. There were some anomalies in the secondary structure classification of these structures, in particular they contained many single amino acid long SSEs. As a result, the length assigned to these proteins was much greater than the realistic number of SSEs. A simple routine removing any of these single amino acid α-helices or β-strands from between linker sections allows these proteins to be located well above the blue minimum bound curve. This cleaning routine was applied to the full sample from the PDB before computing the distribution of acn against the SKMT length. Its effect on the percentage of structures falling below the lower bounding curve was significant as can be seen in S5 Fig. This initial pitfall acts as a reminder of the need to be careful when working with PDB files and secondary structure assignment. Though our initial use for the lower bound on entanglement is for identifying unrealistically folded structural predictions (as highlighted in the main text), it could also serve a purpose in spotting poor secondary structure assignment. This secondary structure cleaning is now performed as standard in the SKMT algorithm, via the simple_ss_clean function. (TIF) [file pcbi.1011248.s005.tif]
