# Supplementary material for: The SKMT Algorithm: A method for assessing and comparing underlying protein entanglement
Source: PLoS Comput Biol. 2023 Nov 27;19(11):e1011248. doi: 10.1371/journal.pcbi.1011248 (PMC10703313; doi:10.1371/journal.pcbi.1011248)
Supplement: S1 Text — The routine used to identify linear subsections of the writhe profile is as follows. First perform a LOWESS (locally weighted scatterplot smoothing) on the writhe data.Then, for all subsections of length greater than 20, compute the gradient of the writhe profile of this subsection.If this gradient is larger than 0.05 (i.e within 50% of the maximally observed linear growth), this subsection is potentially helical.For a potentially helical subsection Ci,j, we then check that there is no change in the sign of the gradient Wr(Ci,i+k)/Wr(Ci,i+j) for all k = 1, j − i. This ensures we identify subsections with consistent linear growth in writhe.Finally, we output the largest disjoint subsections satisfying the above criteria. (PDF) [file pcbi.1011248.s006.pdf]

S1 Text Routine for identifying helical subsections The routine used to identify linear subsections of the writhe profile is as follows.

1. First perform a LOWESS (locally weighted scatterplot smoothing) on the writhe data.

2. Then, for all subsections of length greater than 20, compute the gradient of the writhe profile of this subsection.

3. If this gradient is larger than 0.05 (i.e within 50% of the maximally observed linear growth), this subsection is potentially helical.

4. For a potentially helical subsection $\mathcal{C}_{i,j}$, we then check that there is no change in the sign of the gradient $Wr(\mathcal{C}_{i,i+k})/Wr(\mathcal{C}_{i,i+j})$ for all $k = 1, j - i$. This ensures we identify subsections with consistent linear growth in writhe.

5. Finally, we output the largest disjoint subsections satisfying the above criteria.
