# Supplementary material for: The SKMT Algorithm: A method for assessing and comparing underlying protein entanglement
Source: PLoS Comput Biol. 2023 Nov 27;19(11):e1011248. doi: 10.1371/journal.pcbi.1011248 (PMC10703313; doi:10.1371/journal.pcbi.1011248)
Supplement: Attachment — Submitted filename: review.pdf [file pcbi.1011248.s007.pdf]

The paper defines a number of topologically inspired measures for (open) spatial curves and explores how these measures can be used to analyse proteins' tertiary structures.

While these measures rely on classic knot-theoretical quantities (writhe and average crossing number), the paper presents a number of novelties. Indeed, the authors compute these quantities for all of the subchains of a (discrete) curve, rather than only for the entire object. While analysing sub-chains is not a new idea (especially when analysing proteins spatial configurations), the authors use the evolution of these quantities as the length of the sub-chains increases as a descriptor, which is something new.

Another novelty is given by the model the authors use for proteins. Rather than looking at the piece-wise linear curve given by the spatial coordinates of its amino-acid, the authors start from that curve and obtain a "smoothened" version of it, by applying a modified version of known simplification tools.

The authors then show how they can use this framework to analyse protein structural similarity, to inform predictions of protein structures, and to derive "entanglement related" geometric bounds for the metrics considered.

In my opinion, the techniques developed are novel enough and the results are interesting. The main ideas are quite simple, although they do bring a new angle on topological approaches to study protein structures. Importantly, the quantities used can all be computed quite efficiently, which is not always the case in similar studies. For all of these reasons I do recommend publication.

However, I found the paper's writing quite sloppy in several parts. The introduction is unnecessarily long, and there are several repetitions in the manuscript. I think that the writing should be improved. What follows is a list of specific comments/suggestions.

- Typo in the abstract ("**which and which**")

- Pag 2. **"flat arrows respectively in 1"** missing "figure"

- Pag 9, the fact that the method **"can identify structures which are not classed as similar using standard classification methods"** is repeated twice (once in bold) in a few lines (and also elsewhere in the paper, several times).

- Pag 10, the SWRITHE package is mentioned with no introduction (it is mentioned before in the abstract but I really don't think it is enough)

- Fig 5, caption, I believe **C_in** should be C_1n (which is quite a crucial difference)

- The concept of smoothening is presented in quite a confusing way. In the examples described in the first subsections of the Methods section, quantities are computed on proteins smoothened by taking one amino-acid every three (with no explanation on why this is a reasonable choice), then, we are told that all the remaining computations are performed after a different smoothening procedure, that relies on identifying secondary structures. The authors write that they **"argue a better choice is…"** but they do not argue at all! I would like to see this particular choice to be better justified, or ideally, the authors could even give a quantification (maybe on a portion of the dataset considered?).

- Pag 17, the initial paragraph of **"integer changes and crossing detection"** is badly written. What do they mean by **"when curves change shape"**? I think they mean that the curve changes topological/knot type. If so, they should be precise about it. In general, in this paragraph, I think that certain knot theoretical concepts are described superficially.

- Pag 21: **"The first knot "bound" assumes that the complexity available to a curve/tube increases with length, whilst a linear trend indicates a fixed complexity independent of length, so it seems quite meaningful that such a high percentage fit inside this linear**

trend." It is unclear why it is meaningful, please explain better.

- Pag 21, fix **"Indeed."**

- Pag 23, **"In this way, we see that the writhe measure captures some information that is lost within the knot based measures."** Unclear, explain better.

- The first two paragraphs of the **"Comparing structures using.."** Sections are really difficult to follow. Please rewrite. For example, **"A large number had a length 0 (no structural similarities at all)":** do you mean value instead of length? These typos really make the reading tiring.

More in general, I think that some of the results could be improved/better analysed. Although, I do recognise that perhaps this is beyond the scopes of this project.

- In the results section, the authors discuss two different sets of bounds and notice that the vast majority of proteins discussed fit in those bounds. I would have expected to see (at least some example of) outliers discussed.

- The structural similarity experiment could have been performed in a more systematic way. It seems to be one of the most interesting results/applications of the framework developed, however the idea is tested only on a couple of examples.

Lastly, I think the figures should be done more carefully. In several of them the x-axis labels overlaps with the graph, which is really annoying. Also, fig 17 is hand-drawn, and doesn't look good. Doing the same figure on illustrator or the open-source and freely available Inkscape would take less than half an hour.
