# Supplementary material for: The SKMT Algorithm: A method for assessing and comparing underlying protein entanglement
Source: PLoS Comput Biol. 2023 Nov 27;19(11):e1011248. doi: 10.1371/journal.pcbi.1011248 (PMC10703313; doi:10.1371/journal.pcbi.1011248)
Supplement: Attachment — Submitted filename: ReviewerComments.docx [file pcbi.1011248.s008.docx]

We thank the reviewers for their careful reading of the manuscript and for their comments and constructive suggestions. In response we have significantly reduced the length of the manuscript (by approx. 1 pages) whilst adding additional calculations to justify some of our findings. We have also added a concrete link to the code GitHub/online colab notebook so the reviewers can try the metrics for themselves. We hope the reviewers find this improved paper more focused in its aims and more rigorous in the justification of our findings.

**General Comments in reply:**

The focus of this study is now on the minimal secondary structure smoothing (SKMT method) and how we use it to quantify and compare the complexity of tertiary protein structures. We consider the main findings to be.

1. The bounds on entanglement and their relationship with the secondary structure of proteins obtained using the SKMT algorithm and the use of these bounds to constrain search spaces.
2. The ability of our SKMT based metric to identify large scale helical structures with consistent geometries.
3. The development of a similarity metric based on this SKMT algorithm and its potential to identify previously unexplored relationships between distinct protein families.

Our revisions are aimed at making these arguments as clearly as possible.

Significant additions to the new manuscript include:

1. A justification of the novel SKMT smoothing technique crucial for this work. We have added clear examples which help explain the choice of this method, and why we feel our definition of “length” is an appropriate one. Also, we have added calculations in the appendix of the distributions of writhe obtained for alternative smoothing methods and definitions of length, which are shown not to have the same clear relationships between complexity and length found for the SKMT method, with the aim of solidifying our argument for this choice.
2. An increased focus on tertiary helical geometries as maximal entanglement conformations, and their presence in various sub-domains of protein families. After presenting this work at conferences, we found this aspect of the work was of most interest to the community, with respect to the potential automated identification of Tandem Repeat Domains.
3. A discussion of some of the outliers of the entanglement bounds, with their presence strengthening the relationship between secondary structure and possible entanglement.
4. A discussion as to how these metrics are affected by common issues associated with PDB structures. We highlight a potential problem when working with PDB files and secondary structure assignment, and how this was addressed in this study. Also, in the supplementary material we have addressed the potential for missing termini, including the writhe distribution for our sample with the ends randomly cut. Both add strength to our justification of the SKMT smoothing technique.
5. A more systematic investigation of the similarity comparison metric, including more examples and a study of how the metric depends on the choice of % similarity cut-off.
6. The link to the code is now included in the manuscript. We also provide the dataset of proteins (their SKMT smoothed backbones) used to produce the writhe distributions in this study, along with the code required to perform a similarity sweep for a protein of interest against this database.

With the aim of clarity, we have removed the following aspects from the original manuscript:

1. Sections concerning knotted proteins and net-zero entanglement proteins. These were either not fundamentally new (knotted proteins) or sufficiently justified (net zero) for this more focused manuscript.
2. Removed large parts of the introduction which summarised previous works not fundamental to the content of this revised manuscript.
3. Removed a number of examples and discussions in the properties of the writhe section (e.g Secondary Coiling, Integer Change/Crossing Detection) which have either been discussed in the literature before (and are now cited in the introduction) or relate to the aspects removed in point 1.

One last comment on highlighting our changes. The custom is of course to highlight changes (for example in blue text). However, the changes to the manuscript are so extensive almost the whole document would have to be highlighted if that were the case and it was felt this would negate the aim of the exercise. I lieu of this we have highlighted sections which are genuinely new additions to the manuscript, i.e. the points above. We hope this helps the reviewers asses the more through and systematic arguments we make.

**Specific replies to Reviewer 1**

*“I agree with the authors about the "somewhat lengthy introduction". I think it's nice to give an overview of the topic, but I also think this introduction distracts from the main objectives of the paper. I suggest the authors to try to condense to the key issues.

The style of the intro and of most of the paper gives the reader the feeling that this paper has been put together without much thought, and almost as a descriptive "shopping list" of "stuff" that the authors think are relevant to this topic The title is not very informative
"Novel topological methods for identifying surprising protein tertiary structure relationships." can mean anything. I suggest the authors to convey a bit more precisely their discovery.”*

As discussed above in detail, the introduction has been shortened significantly and we believe the manuscript has a much clearer narrative now. The title has been updated to reflect this.

*“Fig. 9b should have labels on the axes and the plots with the scattering data have no units. Many plots have different style of presentation, font, etc. I'd say that this gives the impression that the paper has been put together in a rush and somewhat unprofessionally.
”*

Figure 9b is no longer included in the updated manuscript. All figures have been updated to use a consistent style.

*“This paper has 29 figures - I am sure that some of them are not necessary and can be put in SI to streamline the discussion. For a reader, especially someone who wants to use their code, it's really hard to understand what is important and what is not.”*

Many figures have been removed or moved to the SI as requested. There are now 17 in the main text.

**Specific replies to Reviewer 2 (PDF)**

*“Typo in the abstract (“which and which”)”*

The typo in abstract removed (with the abstract revised to better reflect the focus of the updated manuscript)

*Page 2. “flat arrows respectively in 1” missing “figure”*

Figure no longer included in revised manuscript.

*“Page 9, the fact that the method “can identify structures which are not classed as similar using standard classification methods” is repeated twice (once in bold) in a few lines (and also elsewhere in the paper, several times).”*

We have endeavoured to remove such repetition in the revised manuscript.

*“Page 10, the SWRITHE package is mentioned with no introduction (it is mentioned before in the abstract but I really don’t think it is enough)”*

The colab notebook is now referenced in the introduction with a link to the github for access.

“*Fig 5, caption, I believe C_in should be C_1n (which is quite a crucial difference)”*

The caption of this figure has been updated to remove this error (our apologies).

*“The concept of smoothening is presented in quite a confusing way. In the examples described in the first subsections of the Methods section, quantities are computed on proteins smoothened by taking one amino-acid every three (with no explanation on why this is a reasonable choice), then, we are told that all the remaining computations are performed after a different smoothening procedure, that relies on identifying secondary structures. The authors write that they “argue a better choice is…” but they do not argue at all! I would like to see this particular choice to be better justified, or ideally, the authors could even give a quantification (maybe on a portion of the dataset considered?)*.”

The method of smoothing unique to this study is now better justified. We have added clear examples which help explain the choice of this method, and why we feel our definition of “length” is an appropriate one. Also, we have calculations in the appendix of the distributions of writhe for alternative smoothing methods/definitions of length. These distributions are shown not to have the same clear relationships between complexity and length found for the SKMT method, with the aim of solidifying our argument for this choice. As for clarity, all figures and calculations in the main text now use the SKMT smoothing. It is now introduced straight away in the methods section, reflective of its increase prominence in the revised manuscript.

*Pag 17, the initial paragraph of “integer changes and crossing detection” is badly written. What do they mean by “when curves change shape”? I think they mean that the curve changes topological/knot type. If so, they should be precise about it. In general, in this paragraph, I think that certain knot theoretical concepts are described superficially. “*

This statement is removed as the reviewer says several papers discuss this issue more clearly and we cite them. Knotting is no longer an explicit feature in the revised manuscript as we believe our novel results/findings are in other aspects of the work/metric.

*“Pag 21: “The first knot “bound” assumes that the complexity available to a curve/tube increases with length, whilst a linear trend indicates a fixed complexity independent of length, so it seems quite meaningful that such a high percentage fit inside this linear trend.” It is unclear why it is meaningful, please explain better.”*

The comment was meant to refer to the fact that a linear increase with length implies longer backbone curves are not accessing the more complex knotting structures implied/permitted by the knotting upper bound of Cantarella et al. However, given we are no longer focussing on knotting in the revised manuscript, we have changed the emphasis of our discussion on this aspect of the findings (the linear growth in writhe for most of the proteins reflective of consistent super-helical geometries).

In the revised manuscript we have removed this sentence and tried to clarify the comment with a more systematic investigation into the helical structures our metric routinely identifies and the fact they dominantly have an increase in writhe with length which is the value of this linear “bound” The choice of the measure of “length” we use is also discussed in more detail by illustrating that other choices (such as the number of points of a uniformly sampled backbone) do not lead to such consistent empirical bounds.

*“Pag 21, fix “Indeed.” “*

Fixed

*“Pag 23, “In this way, we see that the writhe measure captures some information that is lost within the knot based measures.” Unclear, explain better”*

This comment is removed as it is no longer relevant to the revised manuscript’s focus.

*“The first two paragraphs of the “Comparing structures using..” Sections are really difficult to follow. Please rewrite. For example, “A large number had a length 0 (no structural similarities at all)”: do you mean value instead of length? These typos really make the reading tiring. “*

No, although we appreciate the manuscript wasn’t clear enough. It meant there were no sub-sections of the curves with a sufficiently high structural similarity (at some fixed percentage). Some proteins for example have small/medium subsections which are very similar, but some have no subsections at all which are similar, this last set have a length of 0.

The revised discussion on this metric has changed significantly. First, the outcome of the comparison metric depends on a finite cut-off, a % difference between the two structures, dependent on their writhe values, below which we consider them similar. We have now performed a systematic investigation of the effect of this choice by varying this value and showing its effect on the outcome of the metric (the % similarity of the two structures as a whole). Unsurprisingly the lower the required % difference the shorter the percentage of length of the two proteins which are considered similar, but the change in length as a function of this % cut-off is non-linear. We have highlighted visually the effect of this choice to help the reader understand what a particular value implies.

*“More in general, I think that some of the results could be improved/better analysed.*

*Although, I do recognise that perhaps this is beyond the scopes of this project. - In the results section, the authors discuss two different sets of bounds and notice that the vast majority of proteins discussed fit in those bounds. I would have expected to see (at least some example of) outliers discussed. “*

As mentioned above we do now discuss some of the outliers, often (although there is only a small percentage) they are from one of two sources. First, a monomer unit being a simple part of a multimeric complex (so its complexity is only as a part of this complex). Second, from inappropriate secondary structural labelling (e.g., strand helices one amino acid long) which alters the “length” of the curve (this is now automatically remedied by the code). We have given some explicit examples in the revised manuscript and discussed these issues in the text. Increasingly we are finding the bound useful to identify issues in secondary structure labelling in PDB structures (or from DSSP). We consider this a strength of the revised manuscript, so we thank the reviewer for prompting us to investigate this further.

*“The structural similarity experiment could have been performed in a more systematic way. It seems to be one of the most interesting results/applications of the framework developed, however the idea is tested only on a couple of example*s.”

With a view towards addressing this comment we have taken a number of steps. First, the comparison metric depends on a finite cut-off, a % similarity for subsections to be considered similar. We have now performed a systematic investigation of the effect of this choice by varying this value and showing its effect on the outcome of the metric (the % similarity of the two structures as a whole). Second, we have performed a “similarity sweep” of our data base for three structures now. First the Rossman fold/TIM barrel example, second, a knotted protein example (for which we find another un-classified protein which appears nearly identical except for its end terminus threading) and third a “lonely” Rossman fold which matches to an unclassified protein. Whilst this is still not thoroughly “systematic” we feel the distinct nature of the new proteins give the reader a clearer idea of what this comparison measure can tell the user. Third, and perhaps most crucial we have added a functionality to the SWRITHE notebook/code for a user to search a structure against our database. The code for this has been migrated to c++ so it is relatively fast. We hope this will allow the community to find new relationships between proteins such as the Rossmann fold/TIM Barrel structure we have identified.

Finally, we would like in the future to develop some kind of structural of our more generalised notion of similarity to see if any more relationships like the Rossman fold/TIM barrel one highlighted in this paper can be systematically identified. However, we hope the reviewer appreciates this would require significant further study and a would form a paper in its own right.

“*Lastly, I think the figures should be done more carefully. In several of them the x-axis labels overlaps with the graph, which is really annoying. Also, fig 17 is hand-drawn, and doesn’t look good. Doing the same figure on illustrator or the open-source and freely available Inkscape would”*

We have regularised the figures style and endeavoured to remove the issues mentioned. Figure 17 no longer features as it relates to zero writhe structures which, whilst interesting, are not part of the more focused manuscript.

**Specific replies to Reviewer 3**

*“Both smoothing of the backbone prior to calculation of Gauss Integrals as well as a study of their upper bound as function of chain length is not new. It is a major part of Peter Røgen: Evaluating protein structure descriptors and tuning Gauss integral based descriptors 2005 J. Phys.: Condens. Matter 17 S1523*
*Developed precisely (as in the manuscript at hand) to establish relationship at tertiary level (for transition-state ensemble structures) in Protein folding and the organization of the protein topology universe, Kresten Lindorff-Larsen et all. TIBS 30,1 2005”*

The smoothing in this study differs from that in *Rogen: Evaluating protein structure descriptors and tuning Gauss integral based descriptors* both in its method and motivation. The method, as opposed to being locally curvature minimising, is instead a coarse graining based on the secondary structure fingerprint of the protein. The motivation for our smoothing is to reduce the protein backbone to a minimal representation whilst maintaining essential entanglement. It allows the study of the scaling of entanglement as a function of the number of secondary structures. A more detailed justification for this form of smoothing and its relevance to our study is now included in the manuscript. An example now added to the main text highlights the issue: we consider two proteins of roughly equal number of amino acids but one which has a significant number of large alpha helical sections, which are essentially rigid. The complexity is much lower in the protein with the large alpha helical units, reflective of the fact it has less secondary structure sections and hence less potential flexibility (provided by the linker sections). The smoothing in the Rogen paper would, to our understanding, preserve the length of the representative curve (number of points). The crucial issue surrounding our bounds is it is the rise in complexity with respect to the curve’s “length”. Our definition of length, dictated by the smoothing, gives a relatively consistent empirical bound for the protein structures. In the revised manuscript we show numerous other choices don’t.

*“Figure 9a: Why does writhe jump by +-4 at self-intersections? I believe it should be by +-2*
*Michael Livitt used this to detect self-intersections in Protein Folding by Restrained Energy Minimization and Molecular Dynamics, J Mol. Biol. (1983) 170, 723-764“*

There are two simultaneous crossings (arising from the symmetry of the structure). We have removed this example now anyway as it is no longer relevant to the focus of the manuscript, as discussed in detail in the general reply above. We have cited the Livitt paper in the introduction as it was an important oversight.

*The cutting of protein chains into domains (CATH) may vary and for full chains sometimes the N-terminal is missing in pdb-files.The authors therefore need to take into consideration what the deletion of the first 10-20 residues does to all the calculations of writhe (Figures 5, 6 7 10,...) from the beginning to a varying endpoint. What happens if another starting point is chosen?*

This is a good point, to address this we considered randomly cutting 10-20 residues from the start of each protein in our dataset, and then proceeding with the smoothing and writhe calculations from there. The distribution of writhe against number of secondary structures in this case is included in the supplementary information. We found that there is little change in the overall entanglement in this context, a similar fit within the empirical bounds is obtained. Due to the nature of the smoothing used in our method, cutting 10-20 residues would mean cutting 1 or 2 points of the SKMT curve, leading to little change in the overall topology and entanglement scaling.

**Thanks**

**Sincerely**

**The authors**
